# Translating longevity research into healthspan

**DOI:** 10.1186/2046-2395-1-1

**Published:** 2012-09-03

**Authors:** Gordon J Lithgow, Janet M Lord, James L Kirkland

**Affiliations:** 1 Buck Institute for Research on Aging, 8001 Redwood Blvd, Novato, CA, 94945, USA; 2 School of Immunity and Infection, College of Medical and Dental Sciences, University of Birmingham, Birmingham, B15 2TT, UK; 3 Mayo Clinic, 200 1st Street Southwest, Rochester, MN, USA

## 

At the beginning of the 20^th^ century the names of deadly childhood diseases were whispered in fear. Everyone knew a family visited by the shadow of tuberculosis, rickets, polio or smallpox. Chickenpox and rubella brought their own forms of misery. Each disease presented in different ways, affected different organs and had distinct prognoses. Many of us will remember the endless cycles of bake sales and charity runs aimed at raising funds to buy iron lungs for local children badly damaged by polio, and the strings of sanatoriums built for the isolation and treatment of tuberculosis. At the beginning of the 21^st^ century, young parents are scarcely aware of the names of these conditions, never mind stress over their children contracting them. What happened? The germ theory of infectious disease provided the causal framework for the development of Erlich’s magic bullets, Fleming’s antibiotics and Salk’s vaccine. Society didn’t need more iron lungs or sanatoriums; it needed preventions and cures and it got them through investment in microbial, immunological and pharmaceutical research.

At the beginning of the 21^st^ century, the names of different deadly diseases are now whispered. Every family is visited by cancer, Alzheimer’s, Parkinson’s, heart disease, type 2 diabetes or stroke. Other conditions such as arthritis, macular degeneration and generalized frailty bring misery to millions of older adults and their families. Each disease presents itself in different ways, affects different organs and has distinct prognoses. The bake sales and charity runs are still with us; this time it’s not the children they intend to benefit. Our seniors are now the target and there is an urgency to combat these conditions as we are a rapidly aging population. Life expectancy is increasing at approximately two years per decade, with the increase in our elders greatest in the oldest old, the over 85 years group. As a consequence of our failure to develop treatments for the major age-related conditions, the impact of ill health in this age group is significant and growing. Unlike childhood infectious diseases, the chronic diseases of late life are not generally recognized to share a common cause. Their diversity challenges the notion that one or two therapeutic principles could be discovered. But what if there was a common cause to diseases such as cancer and Alzheimer’s? What if “broad spectrum” treatments could be developed? The impact on human suffering would certainly rival the arrival of antibiotics and vaccines and the economic impact would probably be even greater.

The launch of this new journal originates in a belief held by an increasing number of biomedical researchers. The belief is that the chronic diseases of late life do indeed have a single or at least a limited number of causes that can be discovered by studying the mechanisms of normal aging. There is increasing evidence that mechanisms that determine the rate of aging are intimately related to the early events in disease pathology. If this is true, then the notion that a small number of therapeutic approaches, including both lifestyle and pharmacological interventions, might be efficacious against a constellation of age-related conditions. *Longevity & Healthspan* is intended to serve as a venue for this emerging science. Aging and its connection to disease is perhaps the most complex biological problem imaginable and many very difficult challenges lie ahead. Critically, the progress made in understanding factors that determine the rate of aging in animal models has not yet been translated into clinical research. We intend *Longevity & Healthspan* to capture the translational science that should emerge over the next few years.

This journal is being launched at a time of great excitement and optimism in the aging field. The optimism comes from decades of observing healthy lifespan extension resulting from caloric restriction of laboratory rodents. It comes from the ease with which aging can be slowed in invertebrates by genetic and small molecule interventions. It comes from the fine example of successful aging in centenarians. But mostly, the optimism comes from a new generation of scientists who have observed the plasticity of aging with their own eyes and perhaps even intervened in age-related pathology in model systems. This generation of scientists is comfortable working in interdisciplinary teams, an increasingly important skill. Understanding how aging is mechanistically related to disease will require research at the interface of areas of expertise, particularly between basic and clinical scientists.

*Longevity & Healthspan* aims to serve an important role at the interface of basic and clinical aging science. The editorial board is formed from prominent researchers from across a spectrum of basic scientists and clinicians. We expect to publish research from a wide range of disciplines, from model organisms to human clinical studies. We will solicit meeting reports and reviews targeted at emerging areas of interest particularly at the interface of aging and disease. Following peer review, all accepted articles will be free to access online immediately upon publication and in perpetuity, and authors will retain copyright.

We are delighted that BioMed Central has recognized the need and opportunities for a new journal at this time in the development of the aging field and look forward to publishing exciting discoveries in the coming years.

